# MXene Hybridized Polymer with Enhanced Electromagnetic Energy Harvest for Sensitized Microwave Actuation and Self-Powered Motion Sensing

**DOI:** 10.1007/s40820-024-01578-z

**Published:** 2024-11-18

**Authors:** Yu-Ze Wang, Yu-Chang Wang, Ting-Ting Liu, Quan-Liang Zhao, Chen-Sha Li, Mao-Sheng Cao

**Affiliations:** 1 School of Materials Science and Engineering, Beijing Institute of Technology, Beijing, 100081 People’s Republic of China; 2 School of Materials Science and Engineering, Peking University, Beijing, 100871 People’s Republic of China; 3 School of Mechanical and Material Engineering, North China University of Technology, Beijing, 100144 People’s Republic of China; 4 Key Laboratory of Functional Inorganic Material Chemistry, Ministry of Education of the People’s Republic of China, Heilongjiang University, Harbin, 150080 People’s Republic of China

**Keywords:** Microwave absorption, Electromagnetic response, Energy harvest, Self-sensing, Soft actuator

## Abstract

Polymeric microwave actuators combining tissue-like softness with programmable microwave-responsive deformation hold great promise for mobile intelligent devices and bionic soft robots. However, their application is challenged by restricted electromagnetic sensitivity and intricate sensing coupling. In this study, a sensitized polymeric microwave actuator is fabricated by hybridizing a liquid crystal polymer with Ti_3_C_2_T_x_ (MXene). Compared to the initial counterpart, the hybrid polymer exhibits unique space-charge polarization and interfacial polarization, resulting in significant improvements of 230% in the dielectric loss factor and 830% in the apparent efficiency of electromagnetic energy harvest. The sensitized microwave actuation demonstrates as the shortened response time of nearly 10 s, which is merely 13% of that for the initial shape memory polymer. Moreover, the ultra-low content of MXene (up to 0.15 wt%) benefits for maintaining the actuation potential of the hybrid polymer. An innovative self-powered sensing prototype that combines driving and piezoelectric polymers is developed, which generates real-time electric potential feedback (open-circuit potential of ~ 3 mV) during actuation. The polarization-dominant energy conversion mechanism observed in the MXene-polymer hybrid structure furnishes a new approach for developing efficient electromagnetic dissipative structures and shows potential for advancing polymeric electromagnetic intelligent devices.

**Supplementary Information:**

The online version contains supplementary material available at 10.1007/s40820-024-01578-z.

## Highlights

- An alternative electromagnetic attenuation pathway is proposed in the MXene-polymer hybrid structure, distinct from conduction loss, for generalizing the results to a wider range of electromagnetic-thermal driven soft materials and devices.
- By efficiently harvesting and converting electromagnetic energy, the response time of the hybrid polymer to microwave exhibits 87% reduction with merely 0.15 wt% MXene.
- A new mode of self-powered motion sensing based on deformation-driven piezoelectric effect is developed, enhancing the material’s intelligence.

**Supplementary Information:**

The online version contains supplementary material available at 10.1007/s40820-024-01578-z.

## Graphical abstract

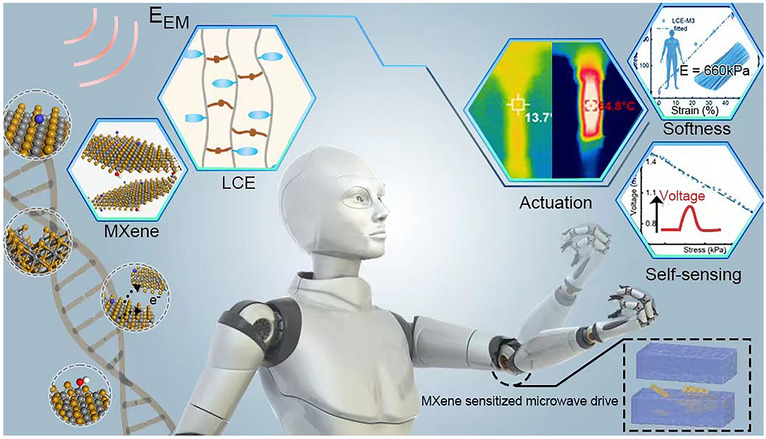

## Introduction

Intelligent polymers have revolutionized the human–machine interaction paradigm and find pioneering applications in bio-robotics, smart clothing, industrial automation and intelligent healthcare [1–3]. Polymeric actuators, including liquid crystal elastomer (LCE), hydrogel, and organogel, represent a novel class of intelligent soft materials that combine the exceptional elasticity of polymers with spontaneous geometric modulation in response to environmental stimuli. Their wide-ranging applications drive advancements in biomass delivery devices, robotic sensors and even new models of energy storage [4–8]. The expanding application scenarios are fueling an increasing demand for polymeric actuators with elevated intelligence. In particular, remote driving and self-sensing are extremely attractive for developing intelligent unmanned devices [9–11].

Wireless energy transmission is an essential prerequisite for remotely driven actuators and other intelligent electromagnetic (EM) devices [12–15]. Currently, most reported strategies for remote actuation of polymeric actuators rely on converting EM energy, such as microwave, infrared and laser radiation, into local heat to trigger thermodynamic conformation transitions. Among these strategies, microwave driving is remarkable for its uniform energy transmission, robustness against obstacles, and fast modulation capabilities, thereby surpassing other forms of actuation strategy in unstructured and enclosed environment [16, 17]. Unfortunately, most polymeric actuators suffer from microwave transparency and exhibit low efficiency in harvesting EM energy from microwaves. To address this issue, polymeric actuators have been hybridized with EM-thermal conversion materials such as liquid metals [18], magnetic particles [19], polar molecules [20], or conductive low-dimensional nanomaterials [21–23], aiming to sensitize their microwave actuation performance. Studies on these hybridized polymeric actuators have revealed that efficient EM energy conversion can be achieved by mechanisms involving polarization loss, magnetic loss or Joule heating, which expedite their microwave actuation response. MXene are an emerging family of two-dimensional transition metal carbides. They provide advantages such as high electrical and thermal conductivity, tunable bandgap and abundant surface functional groups. MXenes have been explored as electromagnetically active material since 2016 [24, 25], and MXene-based EM devices have been reported in numerous cutting-edge studies [26–28]. Nevertheless, the underlying correlation between macroscopic EM properties and microscopic polymer-sensitizer hybrid structures remains unclear. Additionally, the lack of effective dissipative structures for EM energy also leads to excessive use of EM sensitizer, which may challenge the trade-off between microwave sensitivity and desirable mechanical properties including proper modulus, high strength and high energy capacity of the polymeric actuators.

The advancement of stimulus-response sensation can significantly enhance the intelligence level of soft actuators, which is crucial for precise kinetic control and prevents overreactions to external stimuli in natural intelligence. Conventional rigid intelligent devices rely on integrated sensors to acquire sensations, leading to complex electromechanical coupling and desire for energy source module. Various self-sensing approaches based on intrinsic variations have been explored, including electrical resistance variations [9–11, 29, 30], capacitance variations [31], and optical transparency variations [32]. However, these self-sensing prototypes still require supplementary energy supply despite simplifying the sensory system. Recently, neuromorphic computing and artificial synapse technologies have emerged as bionic control systems capable of handling electric signal such as voltage pulse [33, 34]. In this context, a highly appealing prospect is the development of soft actuators that can directly generate electric signals without requiring additional energy input. This capability holds great potential for both bionic intelligent systems and addressing energy challenges. To our knowledge, there are limited studies demonstrating such self-powered feedback capabilities in soft actuators.

To address these challenges, in this study, we develop an MXene hybridized liquid crystal elastomer (LCE-M) that exhibits efficient EM response and sensitive microwave-driven shape memory deformation, the actuation stress of which can further power a polymeric piezoelectric bionic nerve for motion sensing. In such a design, the side-chain LCE is selected as the shape memory matrix due to its reversible contraction from nematic phase to isotropic phase caused by thermodynamic conformation variation. The two-dimensional titanium carbide MXene (Ti_3_C_2_T_x_) known for its diverse surface chemistry and tunable dielectric properties, is hybridized with the LCE matrix through intermolecular interaction such as hydrogen bonding. Investigation into EM energy conversion in the MXene-polymer hybrid structure reveals that versatile polarization-relaxation processes dominate dielectric loss and heat generation, distinguishing it from conventional conduction loss or Joule heating mechanism. Consequently, by varying the loading content of MXene at an ultra-low level, both sensitized microwave actuation performances and mechanical properties of the hybridized polymer can be tuned accordingly. With a loading content of 0.15 wt%, the hybridized smart polymer demonstrates an 8.3 times higher apparent EM energy harvest efficiency compared to the pure LCE, resulting in an 87% reduction of response time to microwave irradiation. The improved work capacity of the hybrid polymeric actuator also enables mechanical–electrical energy conversion using a piezoelectric nerve made from polyvinylidene fluoride (PVDF) operating in d_31_ mode. A concurrent millivolt magnitude piezoelectric voltage can be detected alongside LCE-M’s contraction, providing motion sensation without requiring additional energy input, and showcasing potential applications for fully compliant polymeric intelligent devices.

## Experimental Section

### Materials

Ti_3_AlC_2_ powder (300 mesh) was obtained from Shandong Xiyan New Material Technology Co., Ltd. Hydrochloric acid (35 wt%) and LiF was purchased from Macklin Technology Ltd (Shanghai, China). Polydimethylhydrosiloxane (PMHS) was obtained from ACROS Chemicals (Belgium, USA). Commercial platinum catalyst Pt(COD)Cl_2_ was purchased from Aldrich (St Louis, USA). Polyvinylidene fluoride (PVDF, MW ~ 20,000) and dimethyl formamide (DMF, 99%) was supplied by Macklin Technology Ltd (Shanghai, China). All reagents were of analytical grade and used without further purification.

### Preparation of Ti_3_C_2_T_x_ and LCE-M Actuators

#### ***Preparation of Ti***_***3***_***C***_***2***_***T***_***x***_

Fewer-layer Ti_3_C_2_T_x_ was exfoliated from Ti_3_AlC_2_ powder through modified HF etching method. LiF (1 g) was dispersed in 6 M HCl (20 mL), followed by dispersing Ti_3_AlC_2_ powder (1 g) into the mixture. The etching process lasted for 24 h under 50 °C, after which the product was washed with deionized water until its pH was more than 6. Then the product was centrifugated at 3,500 rpm for 10 min, and the supernatant was further centrifugated at 7,000 rpm for 10 min after violent shaking. The result supernatant was collected and freeze-dried to obtain Ti_3_C_2_T_x_ powder.

#### Preparation of LCE-M Actuators

Mesogenic units 4-methoxyphenyl-4-(1-buteneoxy)benzoate (MBB) and cross-linker molecule 1,4 alkeneoxybenzene (11UB) were synthesized by modifying synthetic route in concerned papers, as is shown in Fig. S1. Their chemical structures were characterized by ^1^H-NMR spectroscopy before use (Fig. S2). PHMS (48 mg), MBB (200 mg) and 11UB (27.2 mg) co-dissolved in 0.8 mL of toluene. Platinum catalyst (40 μL, 1.25 mg mL^−1^) was injected to the reaction system in a Teflon mold (4 cm × 1.2 cm × 1 cm) before reaction. The sealed mold is then heated at 65 °C for 40 min, followed by stripping the resultant cross-linked elastomer from the mold. In ambient condition, adsorbed solvent evaporated and the elastomer obtained a stable length after gradual shrinkage. Mechanical stretching was conducted by loading a weight (~ 5 g) to the primary cross-linked elastomer. After getting stable in a fixed length, the elastomer was treated at 70 °C for 12 h for secondary cross-linking. The pure LCE was obtained after cooling it down to room temperature. MXene implantation was realized by dispersing the freeze-dried Ti_3_C_2_T_x_ powder in reaction solution mentioned above with controlled mass ratio (0.05%, 0.1%, and 0.15%), resulting in LCE-M1, LCE-M2, and LCE-M3, respectively. Note that Ti_3_C_2_T_x_ was dispersed previously in toluene and was ultrasonic treated for 5 min before use.

### Characterizations

#### Structural Characterizations

Scanning electron microscopy (SEM, HITACHI S-4800) and field emission transmission electron microscopy (TEM, FEI Talos F200S) were employed to characterize the morphology of Ti_3_C_2_T_x_ and LCE-M composites. X-ray powder diffraction (XRD) was collected from X-ray diffraction (XRD, X’Pert PRO) using Cu-Ka radiation. Two-dimensional wide-angle X-ray scattering (2D-WAXS) was performed by a 2D X-ray diffractometer (Bruker Nanostar). Polarizing optical microscopy (POM) (SMZ1500, Nikon Instruments) was employed to determine the light anisotropy of LCE matrix. ^1^H-NMR spectra were recorded on Bruker 400 MHz at 20 °C. Young’s moduli of the samples were collected by a dynamic mechanical analyzer (Q800 DMA, TA Instruments). Raman spectrum was recorded by a Raman microscope (Horiba Scientific) with a 514-nm laser beam as the light source. ATR-FTIR spectrum was collected by a Nicolet iS20 spectrometer (Thermo Scientific).

#### Characterizations of EM Properties and S-Parameters

EM properties and S-parameters are measured using a vector network analyzer (VNA, Anritsu 37269D) by coaxial method from 2 to 18 GHz at room temperature. The samples were punched into a toroidal shape (Φouter = 7.03 mm; Φin = 3.00 mm) for testing. Differential scanning calorimetry (DSC, TA Instruments Q100 modulated differential scanning calorimeter, New Castle, DE) measurements were conducted at heating rate of 10 K min^−1^ under nitrogen flow to verify the phase transition behaviors of the samples.

#### Characterizations of Microwave Actuation Performance

Actuation strain tests were carried out by a homemade deployment as shown in Fig. S10. TOSER-MP1000SL microwave generator was employed in microwave actuation performance test, which is carried out at a room temperature of 12 °C. Two reflectors were employed to confine the electromagnetic field. The metal basket was used to make up for the hole of larger reflector where the monitor was deployed. Infrared thermal images were collected with an infrared thermal imager (Sigma ST9660). The actuation performances of LCE-M actuators were monitored by an infrared laser range sensor (QAD-2800).

Actuation stress was tested in fixed length mode using the dynamic mechanical analyzer (Q800 DMA, TA Instruments). The piezoelectric voltage was monitored by a digital voltmeter (Victor 8246BZ020003, Xi’an Victor Instruments).

## Results and Discussion

### Fabrication Strategy and Structural Characterizations

**Fig. 1:**
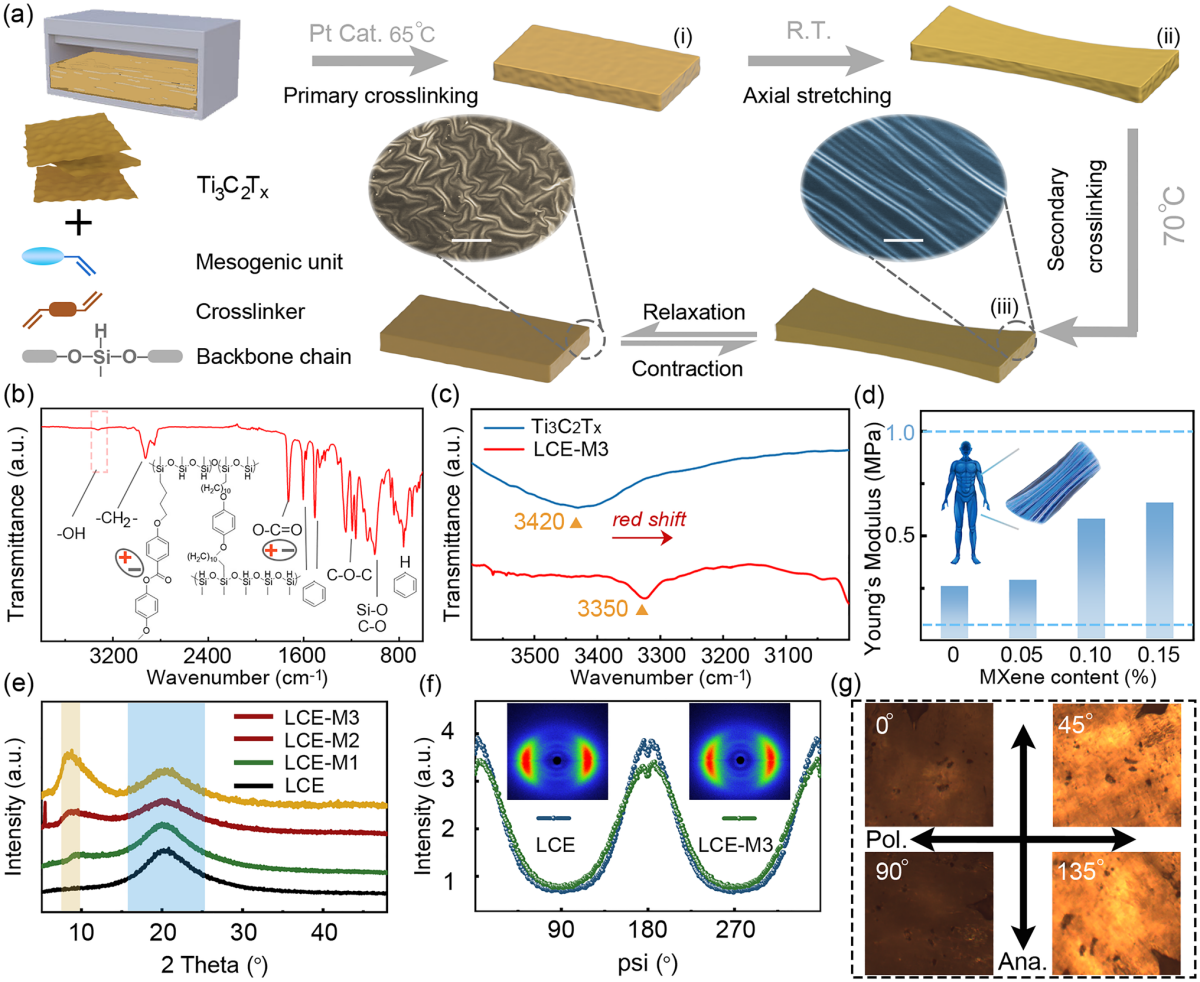
Preparation and characterization of LCE-M. **a** Schematic illustration of the preparation procedure for LCE-M; Insets in **a** are the SEM images showing isotropic conformation (left) and nematic conformation (right) of LCE-M in cross-sectional view (scale bar, 10 µm). **b, c** FTIR spectrum of the LCE-M hybrid and Ti_3_C_2_T_x_. **d** Comparison of Young’s modulus of LCE and LCE-M samples. **e** XRD characterizations for LCE-M samples. **f** Azimuthal diffraction profiles for LCE and LCE-M3 and their corresponding 2D-WAXS patterns (inset). **g** POM images of LCE-M3. Angles inset indicate the deflection of sample to analyzer

The LCE-M hybrid polymer is synthesized through a two-step polymerization strategy, as illustrated in Fig. 1a. In the first step, the freeze-dried MXene is dispersed in the precursor solution of LCE containing mesogenic unit (MBB), cross-linker (11UB) and backbone chain (PHMS). Covalent bonding between these components is achieved via Si–H addition reaction (Figs. S1–S3). During primary cross-linking, a lightly cross-linked structure is formed with an expected cross-linking density of 16% [35]. No discernible optical anisotropy is observed by polarizing optical microscope (POM) at this stage, indicating the internal polymeric network remains isotropic (Fig. S4). Subsequently, mechanical force exceeding the internal stress is applied along the axial direction to stretch the matrix to ~ 125% of its original length. This mechanical stretching induces alignment of mesogens and leads to a temporary anisotropic conformation of the polymer. The liquid crystal order provided by mesogenic MBB hanging over PHMS backbone chain is fixed through complete covalent bonding during secondary cross-linking. The reversible macroscale deformation of the hybrid polymer arises from thermodynamic variations between nematic and isotropic conformations (inset in Fig. 1a).

Intermolecular interactions between LCE matrix and MXene facilitate the stability of the MXene-polymer hybrid structure. SEM images in Fig. S5 show that MXene sheets are distributed throughout the matrix with steady implantation states. Compared to their morphology before hybridization with LCE (Fig. S5d, h), implanted MXene sheets present thicker edges due to the polymer layer attached to their surface. The attenuated total reflectance Fourier transform infrared spectroscopy (ATR-FTIR) of LCE-M3 in Fig. 1b characterizes the molecular structure of the LCE matrix with polar groups such as ether group and easter group. A weak absorption band is observed at ~ 3350 cm^−1^, corresponding to surface hydroxyl on MXene. The redshift of this band compared to 3420 cm^−1^ for pure MXene suggests intermolecular hydrogen bonding between LCE and MXene (Fig. 1c) [36]. As shown in Figs. 1d and S6, an increase in MXene content results in higher Young’s modulus for LCE-M actuators, further proving intermolecular interaction between MXene sheets and LCE matrix. Notably, all LCE-M samples exhibit moderate modulus in the range of 0.1–1 MPa benchmarking softness against tissues like muscle [37]. The XRD pattern of LCE in Fig. 1e shows a wide peak at ~ 21° due to the limited crystallinity of its polymeric network. After hybridizing with MXene, the characteristic diffraction peak of (002) lattice plane of MXene appears at ~ 7.8° [38], and its intensity rises with higher MXene content. The half-peak breadth of the peak at ~ 21° mildly increases without position shift, suggesting decreased crystallinity of LCE after hybridizing with MXene. The above results prove effective hybridization between MXene and LCE.

The nematic conformation of the LCE-M hybrid structure is investigated through wide-angle X-ray scattering (2D-WAXS) and POM characterizations. Two crescent-like scattering patterns are observed in 2D-WAXS plots with and without MXene hybridization, indicating the nematic alignment of mesogens for both LCE and LCE-M3 (Fig. 1f inset). Order parameters (*S*) are determined based on 2D-WAXS results (Fig. 1f), using the azimuthal diffraction intensity (Eqs. S1 and S2) [39]. The *S* value for LCE-M3 is found mildly lower at 0.227 compared to pure LCE at 0.255, indicating a slight decrease in the nematic alignment degree. Figure 1g presents the POM images of LCE-M3 under different angles to the crossed optical polarizers. The bright view of birefringence at 45°/135° also confirms the presence of a nematic conformation in mesogenic LCE-M3 as well. These findings indicate that despite mild reduction in mesogen alignment due to intermolecular interaction with MXene, the nematic phase is maintained in the LCE-M hybrid structure.

**Fig. 2:**
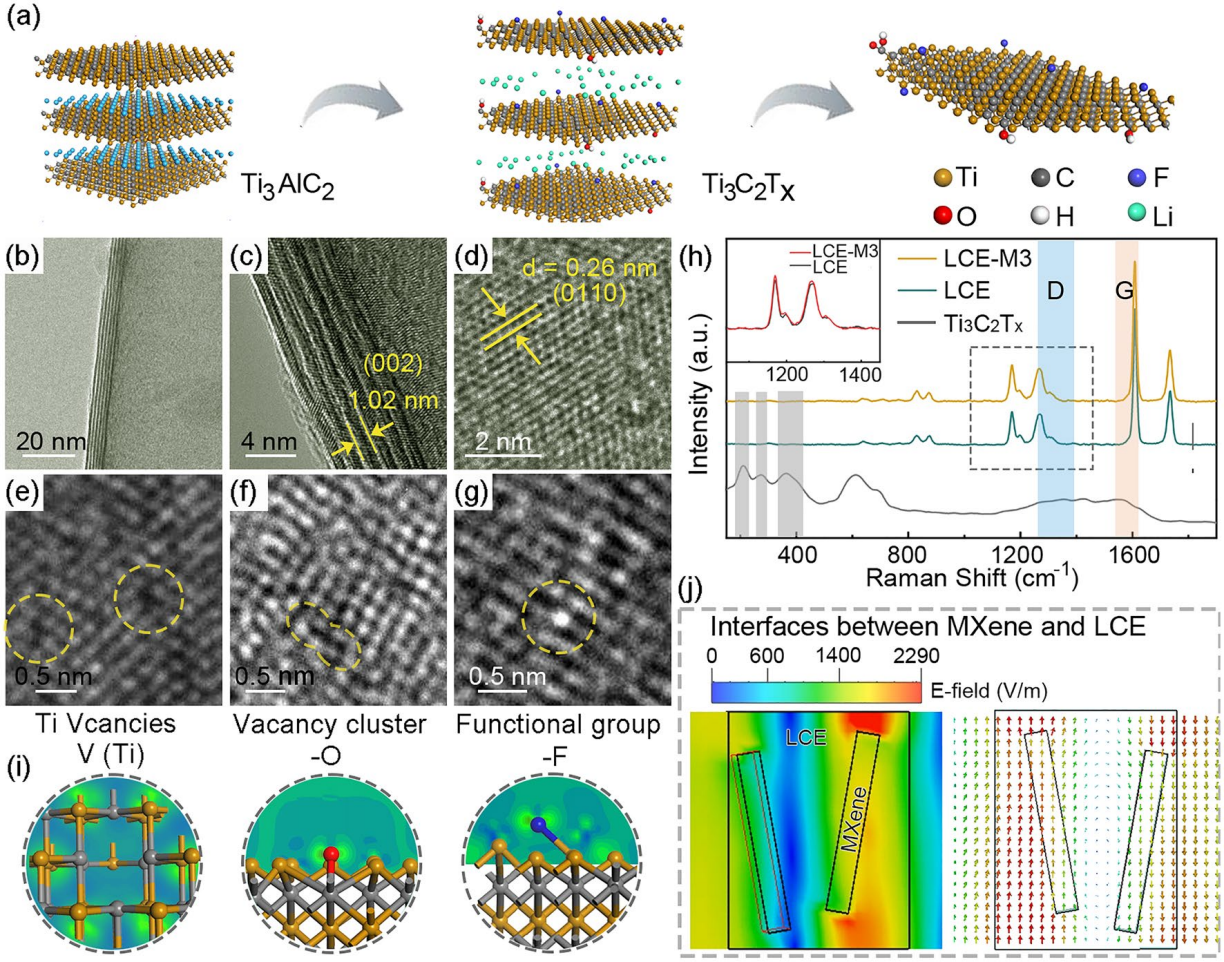
Characterization of the polarization “genes” in LCE-M. **a** Schematic illustration of the chemical etching process of Ti_3_C_2_T_x_ nanosheet. **b**–**d** HR-TEM images of Ti_3_C_2_T_x_ in different magnifications. **e**–**g** Atomic-scale HR-TEM images presenting defects in Ti_3_C_2_T_x_. **h** Raman characterization for Ti_3_C_2_T_x_, LCE and LCE-M. **i** Charge density difference simulation for the defect areas in Ti_3_C_2_T_x_. **j** Simulation of *E*-field between MXene sheets and LCE matrix

Further structural characterization reveals the dielectric “genes” inherent in the LCE-M hybrid structure, which determine its electromagnetic response. As the crucial electromagnetically active component, Ti_3_C_2_T_x_ nanosheets are derived from Ti_3_AlC_2_ (MAX phase) and form diverse polarization centers at the surface defect areas (Fig. 2a). The high-resolution transmission electron microscope (HR-TEM) image in Fig. 2b shows the structure of few-layer Ti_3_C_2_T_x_, with an inter-layer distance measuring 1.02 nm corresponding to the expanded lattice spacing of the (002) plane (Fig. 2c) [40]. Clear lattice fringes observed in Fig. 2d exhibit a lattice spacing of 0.26 nm that corresponds to the 0110 plane of Ti_3_C_2_T_x_ lattice [41, 42]. Atomic-scale HR-TEM observation reveals defects and lattice distortion on Ti_3_C_2_T_x_ sheets as shown by Fig. 2e–g. Intrinsic defects including Ti vacancies and C vacancies can be introduced during exfoliating the Ti_3_C_2_ layer. Meanwhile, removal of intercalated Al layers between Ti_3_C_2_ results in unsaturated Ti atoms at defect areas partially bonding with adsorbed oxygen, fluoride ion and hydroxyl groups during etching, leading to the out-of-layer adatoms and terminal functional groups including –O, –F, and –OH [43]. Raman spectra further characterize the defects in Ti_3_C_2_T_x_ as depicted in Fig. 2h. Three distinct Raman peaks appearing from 180 to 360 cm^−1^ correspond to different directions of Raman-active photon vibration termed as *ω*1, *ω*2, *ω*3, and *ω*4 [44]. In comparison with pristine Ti_3_AlC_2_ material, the peaks of *ω*1 and *ω*4 are broadened (Fig. S7), which indicates the formation of adsorbates and derived functional groups during chemical etching [45]. The characteristic G band at 1600 cm^−1^ and D band at 1350 cm^−1^ can be attributed to partial exposure and distortion of sp^2^ carbon layer, which suggests the existence of surface Ti vacancy clusters. LCE-M3 exhibits similar Raman spectra compared to pure LCE due to the ultra-low content of Ti_3_C_2_T_x_. A mild increase recorded at ~ 1350 cm^−1^ is due to the overlap of D band of Ti_3_C_2_T_x_ as shown by the inset.

The defects and functional groups are investigated through first principles calculations to simulate the electron density distribution. As depicted in Fig. 2i, the presence of vacancy defects and polar functional groups on Ti_3_C_2_T_x_ leads to uneven charge distribution at the local sites, enabling them to capture mobile charge carriers excited by external electric fields. When subjected to oscillating EM field, the abundant inherent vacancies and terminal functional groups serve as inherent polarization centers, resulting in space-charge polarization. In addition to the atomic-level polarization centers inherited from MXene, polar groups within the LCE polymeric network and the heterointerface between MXene and LCE further diversify the dielectric “gene” within the LCE-M hybrid structure. Figure 2j and Movie S1 illustrate non-uniform electric field distribution at the heterointerface between LCE matrix and MXene sheets. Interface dipoles are generated as a result of the charge capacity variance across both sides of the heterointerface, leading to relaxation loss of EM energy when their orientation lags behind the external EM field oscillations [46]. Therefore, MXene hybridization introduces a wealth of polarization genes into the LCE-M hybrid structure that can effectively modulate its EM response.

### Relaxation “Genes” Dominant Electromagnetic Energy Conversion

**Fig. 3:**
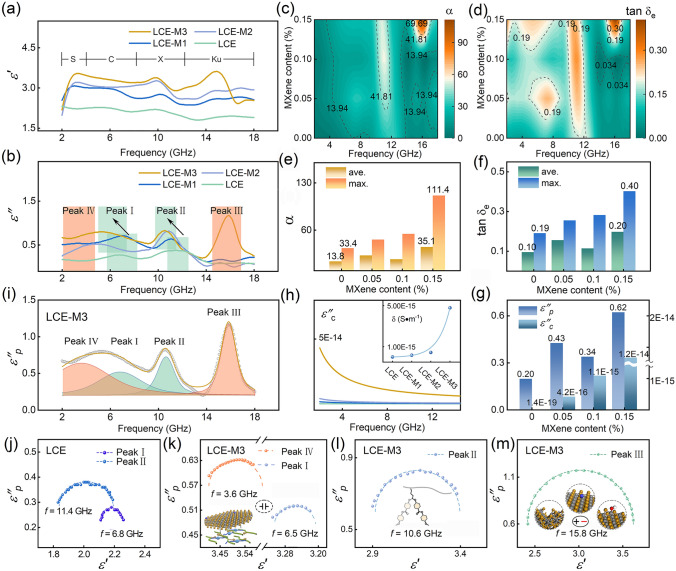
Dielectric properties characterization for LCE-M hybrid polymers. **a**, **b** Complex permittivity and **c**, **d** Counter maps of attenuation constant (*α*) and loss tangent (tan*δ*_e_) versus MXene content in 2–18 GHz. Assessment for **e** *α* and** f** tan*δ*_e_ in the investigated frequency band. **g** Assessment for polarization loss (*ε″*_p_) and conduction loss (*ε″*_c_) in the LCE-M samples. **h** *ε″*_*c*_ in LCE-M samples and their fitted conductivities. **i** Deconvolution of relaxation peaks based on *ε″*_*p*_ in LCE-M3. **j** Cole–Cole plots derived from LCE in the frequency range corresponding to peak I and peak II. Cole–Cole plots derived from LCE-M3 in the frequency range corresponding to: **k** peak I and peak IV; **l** peak II and **m** peak III

The dielectric property of LCE-M hybrid structure is investigated to elucidate its EM response and the EM energy harvesting capacity in specific microwave band (2–18 GHz). As shown in Fig. 3a, b, both the real part (*ε′*) and imaginary part*″* (*ε″*) of the complex permittivity increase with the MXene content in LCE-M, indicating a dielectric enhancement effect due to MXene hybridization. The stepwise decline in *ε′* and corresponding peaks in *ε″* appearing with increasing frequency suggest a polarization-relaxation process within the material. Pure LCE exhibits relaxation peak I and II at C and X bands, attributed to relaxation processes based on polar molecules within the polymeric network. In contrast, for the LCE-M hybrid structure, peak III appears at Ku band while peak IV appears at S band, and their intensities increase with higher MXene content (Fig. S8). Additionally, there is a redshift observed for relaxation peaks I and II in the LCE-M hybrid structure, resulting in peak I merging with peak IV to form a broad peak (Fig. 3b). This redshift suggests prolonged relaxation time of molecular orientation polarization, due to the hysteresis effect caused by Ti_3_C_2_T_x_ sheet on molecular chain swinging. The dielectric loss capacity is further characterized by loss tangent (tan*δ*_*e*_) and attenuation constant (*α*), which exhibit an increasing trend with higher MXene content (Fig. 3c, d). Notably, LCE-M3 demonstrates an average tan*δ*_*e*_ that is 107% higher than that of pure LCE along with an average *α* that is 153% higher compared to LCE (Fig. 3e, f). These results indicate the efficient enhancement of dielectric loss capacity and EM response across a widened microwave band achieved through incorporation of MXene into the LCE matrix.

Typically, *ε″* represents the loss capacity to EM energy, comprising relaxation loss (*ε″*_*p*_) and conduction loss (*ε″*_c_). These two factors are distinguished from *ε″* to assess different EM energy conversion mechanisms in the LCE-M hybrid structure. As depicted in Fig. 3g, *ε″*_*p*_ exhibits orders of magnitude higher values than *ε″*_c_ in all the four samples, indicating the relaxation loss contributes the major proportion of dielectric loss in the LCE-M hybrid polymer. Figure 3h demonstrates an increased value of *ε″*_c_ and conductivity with higher MXene content in LCE-M. Moreover, the magnitude of conductivity shows a linear dependence on MXene content (Fig. S9), suggesting that conductive MXene sheets contribute to local conductance but do not form a conductive network through the matrix due to their ultra-low content.

According to Debye’s relaxation theory and the circular arc law (Eqs. S3-S5), a classic relaxation process should exhibit a semicircle curve in the Cole–Cole plot. By performing deconvolution fitting, four relaxation peaks are observed in *ε″*_*p*_ of LCE-M3 across S to Ku band (Fig. 3i). Figure 3j shows Cole–Cole circular arc observed in the initial LCE sample, corresponding to the orientation polarization of polar molecular chain (peak I and II). Figure 3k–m illustrates Cole-Cole plots corresponding to the four relaxation peaks from S to Ku band in LCE-M3. These well-fitted circular arcs indicate the relaxation processes conform to Debye’s theory. In addition to the orientation polarization associated with redshifted peaks I and II, peak III relaxation in LCE-M3 at high-frequency band can be attributed to space-charge polarization induced by abundant functional groups and surface defects on Ti_3_C_2_T_x_ (Fig. 3m inset). Relaxations at lower frequency bands are primarily associated with slow polarizations such as interface polarization [47, 48]. The presence of capacitor-like heterointerface as discussed above between Ti_3_C_2_T_x_ sheet and polymer matrix contributes to interface polarization (Fig. 3k inset), as evidenced by exhibiting Cole–Cole circular arc in S band (Fig. 3k). In summary, polarization-relaxation loss is identified as the dominate mechanism for EM energy attenuation in LCE-M hybrid structures, which can be finely tuned at atomic, molecular and intermolecular levels, thereby providing a pathway for broadband EM energy conversion distinct from conventional conduction loss or Joule heating. The contribution to EM energy absorption is also proved by elevated specific absorption efficiency (*SSE*_*A*_) as depicted in Fig. S10. With an increased MXene content in LCE-M, the *SSE*_*A*_ exhibits a corresponding enhancement, indicating an improved proportion of absorbed EM energy through the hybrid structure.

### Microwave Actuation Performance

**Fig. 4:**
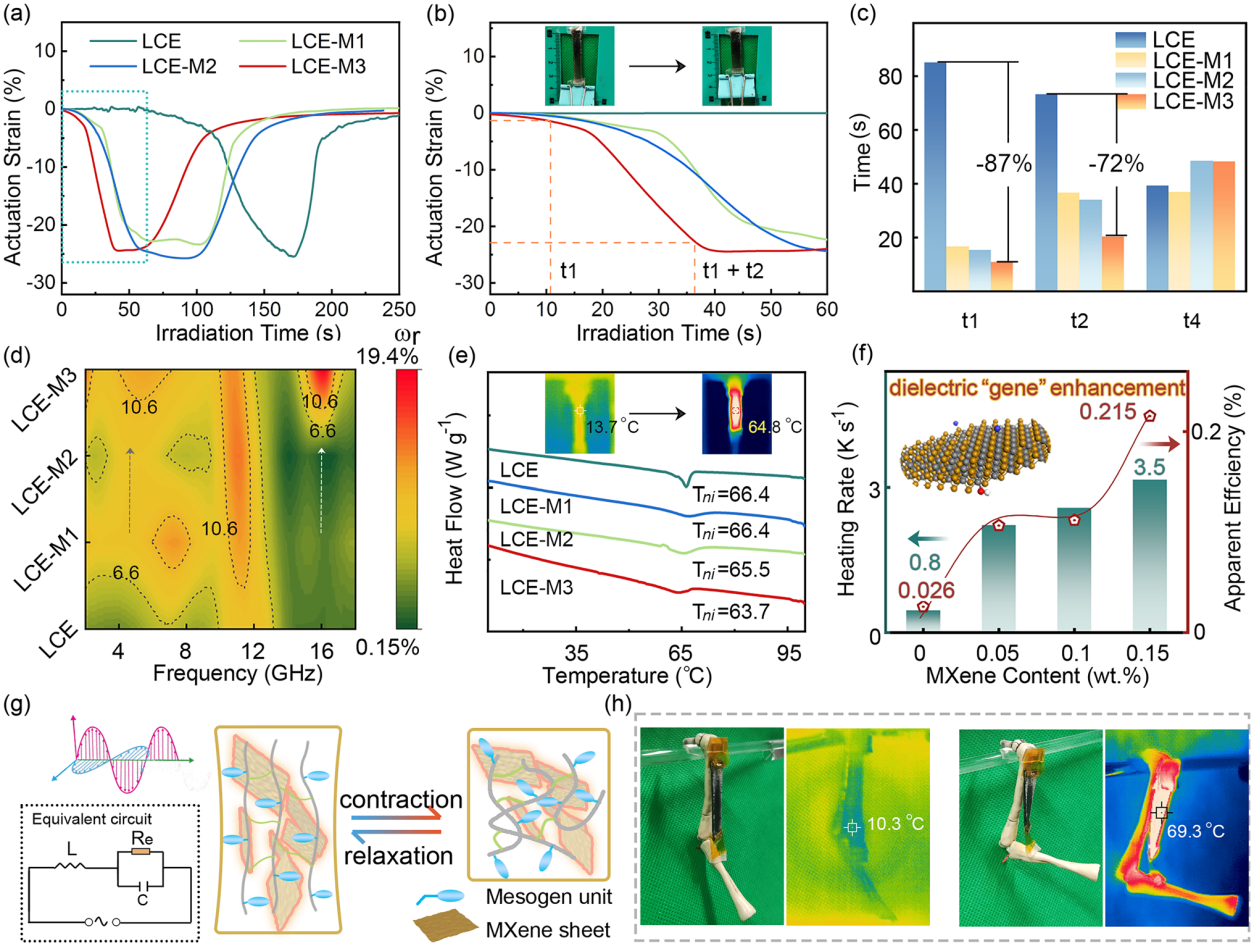
Microwave actuation performance of LCE-M hybrid polymer. **a**, **b** Actuation strain of the four samples under microwave irradiation versus time. Inset in **b** is the photographs depicting the microwave-induced deformation of LCE-M3. **c** Comparation of *t*_*1*_, *t*_*2*_ and *t*_*4*_ of the four samples in the microwave actuation process. **d** *ω*_*r*_ of LCE-M actuators versus frequency. **e** DSC analysis for LCE-M samples. Insets in **e** are the infrared thermal images corresponding to the insets in **b**. **f** Assessment of the heating rates and apparent EM energy harvest efficiencies under microwave radiation. **g** Microwave driving mechanism with an equivalent circuit of the LCE-M actuator under microwave irradiation. **h** Photographs depicting the remote control capabilities of a microwave-driven muscle-like LCE-M actuator and the corresponding infrared thermal images

To verify the sensitization effect of microwave actuation performance, LCE-M-based actuators are tested in a homemade device consisting of a microwave source (2.45 GHz), reflector, visible and infrared thermal monitor, as well as a laser ranging device (Fig. S11). Under 500 W (rated output power) microwave irradiation, the LCE-M actuators (30 mm × 4 mm × 1.2 mm) exhibited reversible anisotropic uniaxial contractions that return to its initial length upon removal of the microwave stimulus (Movie S2). Figure 4a, b illustrates the actuation strain of LCE-M actuators versus microwave irradiation time. The maximum strain observed for LCE, LCE-M1, LCE-M2, and LCE-M3 are found to be 26.0%, 24.6%, 26.0%, and 25.5%, respectively. The deformation period of the actuators following exposure to microwave can be divided into four parts: (i) response period (time from microwave activating to 5% of maximum strain); (ii) deformation period (time from reaching 5% to reaching 95% maximum strain); (iii) period of complete deformation and cooling without microwave; (iv) period of geometric recovery with shape memory effect. Obviously, the sensitivity of microwave actuation can be assessed by examining the response period (*t*_*1*_) and deformation period (*t*_*2*_), while the recovery period (*t*_*4*_) is associated with mechanical properties and shape memory effect of the actuator (Fig. S12). Figure 4c evaluates the *t*_*1*_, *t*_*2*_, and *t*_*4*_ for all actuators. Importantly, MXene hybridization significantly enhances the sensitivity of microwave-induced deformation in LCE-M3 with a remarkable actuation (5% of maximum strain) achieved within only 10.9 s (*t*_*1*_), representing an impressive decrease by 87%. Additionally, the complete deformation is attained within 20.3 s (*t*_*2*_), demonstrating a substantial reduction by 72% compared to pure LCE. Moreover, the increase in *t*_*4*_ suggests that MXene hybridization leads to suboptimal shape memory effects due to the diluted alignment degree of mesogens (Fig. 1f). Therefore, an ultra-low filler loading is pivotal and MXene content in LCE-M is up to 0.15% since a significant improved EM energy harvesting efficiency is achieved. In comparison to previously reported polymer-based actuators responding to microwave, LCE-M hybrid polymer stands out due to its remarkably low content of EM sensitizer, realizing 87% reduction in response time with merely 0.15% MXene hybridization (Table S1).

The issue of EM energy conversion underlying sensitized microwave actuation is further analyzed. It is accepted that the EM energy absorbed by dielectric material can either be stored in the material or converted to thermal energy that dissipates [49–51]. Based on the inset equivalent circuit model shown in Fig. 4g, the ratio of converted EM energy (*E*_c_) to stored energy (*E*_s_) in the LCE-M hybrid structure can be evaluated using Equation S6. As depicted in Fig. 4j, compared with unsensitized LCE, LCE-M demonstrates an improved converted ratio. The *ω*_*r*_ of LCE-M3 is 9.4 and 3.4 times higher than that of LCE at 16.2 and 2.4 GHz, respectively, indicating a significant improvement in converting EM energy to heat due to the space-charge polarization and interface polarization within the hybrid structure. This observation is also supported by infrared thermal images showing deformation of LCE-M accompanied by temperature rise under a 2.4 GHz microwave stimulus (inset in Fig. 4e). Differential scanning calorimetry (DSC) analysis reveals the thermodynamic actuation threshold of LCE-M, as shown in Fig. 4e. With increasing MXene content, the phase transition temperature (*T*_*ni*_) gradually decreases from pure LCE of 66.4 °C to that of LCE-M3 which is measured at 63.7 °C, indicating that MXene hybridization significantly reduces the thermal threshold for microwave actuation. Figure 4f illustrates how heating rate increases with the MXene content, ranging from a heating rate of pure LCE at only 0.6 K s^−1^ up to LCE-M3 at a much faster rate of approximately 4.7 K s^−1^. The apparent EM energy harvest efficiency (*AE*_*EM*_), which refers to the proportion of converted EM energy to the rated output, is evaluated versus MXene content using Eqs. S8-S10. The *AE*_*EM*_ of LCE-M actuators increases with a higher content of MXene, and the *AE*_*EM*_ of LCE-M3 dramatically increases by 8.3 times compared to that of pure LCE. Therefore, the sensitization mechanism of microwave actuation can be summarized as follows: The dielectric loss originating from diverse polarization-relaxation processes enhances EM energy harvesting and its conversion to heat, inducing a rapid temperature rise in LCE-M actuator. Meanwhile, the hybridized MXene reduces the thermodynamic threshold of phase transition. Under this synergetic effect of sensitization, an accelerated thermodynamic variation between nematic conformation and isotropic conformation leads to macroscale deformation (Fig. 4g). Considering the marginal decrease in actuation threshold with increasing MXene content, the dominant factor sensitizing the actuation of LCE-M actuators is relaxation-induced EM energy conversion and heat generation.

The LCE-M shows long-term stability and its repeatability is validated by repetitively inducing microwave-responsive deformation over an ambient storage period of 8 days. As depicted in Fig. S13, LCE-M3 maintains its sensitivity and deformation capability upon exposure to microwave irradiation, which can be due to the inherent stability of the Si–O rubbery matrix [52, 53] and its protection effect on hybridized MXene against degradation. Corresponding polarizing optical microscope (POM) results proved that the nematic conformation of LCE-M was preserved over the period. To demonstrate the remote control potential of LCE-M actuators in microwave actuation mode, a bionic arm is constructed where LCE-M actuator mimic the muscle function, as is shown in Fig. 4h and Movie S3. Infrared thermal images confirm successful remote energy transfer achieved through microwave absorption. The actuation stresses of LCE-M actuators are examined under fixed length conditions (Fig. S11). Increasing MXene content in LCE-M hybrid polymers results in higher maximum actuation stress, consistent with the trend observed for Young’s modulus (Fig. S14). The maximum actuation stress for LCE-M3 is 75 kPa. According to Eq. S11, the work capacities of LCE-M actuators range from 7.2 to 15.5 kJ m^−3^. Notably, this surpasses the average level observed in skeletal muscles (~ 8 kJ m^−3^) [37, 54]. Taking the deformation period into account, the power density of LCE-M3 reaches 525 W m^−3^ (Eq. S12). The exceptional performance of this hybrid polymer in terms of reversible actuation, proper elasticity and softness makes it a promising candidate for developing all-polymer-based self-powered deformation sensing.

### Self-Powered Motion Sensing

**Fig. 5:**
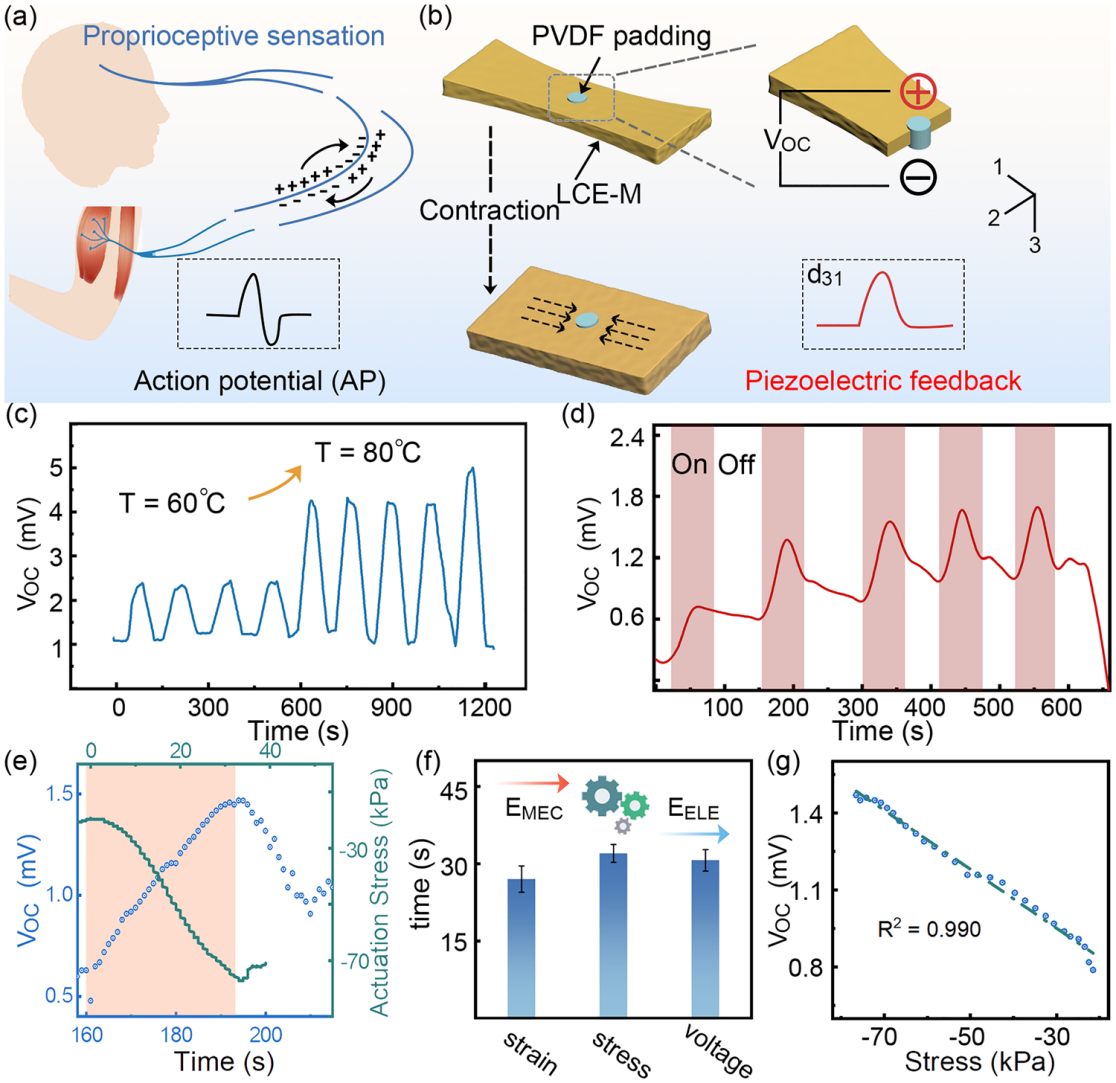
Self-powered motion-sensing capability of LCE-M-based polymeric device. **a**, **b** Schematic comparison in motion sensing between the mammalian motion system and LCE-M-based piezoelectric motion sensing. *V*_*OC*_ output collected under: **c** different temperature and **d** cyclic 250 W light irradiation. **e** *V*_*OC*_ and actuation stress versus time during light-driven deformation. **f** Comparison of response time observed from contraction strain, stress and piezoelectric voltage. **g** Linear dependance of the* V*_*OC*_ on the actuation stress. Sample size for error bars: 5

In the mammalian motion system, the sensory nerve generates motion feedback from muscle contraction through the sensory nerve action potential (SNAP), which relies on variations in membrane potential, as depicted in Fig. 5a. Following a biomimetic approach, LCE-M3 actuator is equipped with a self-powered actuation sensing capability using piezoelectric PVDF polymer. A schematic illustration of this device is presented in Figs. 5b and S15. Initially, a circular hole (*φ* = 3 mm) was punched into the LCE-M3 actuator, which was then filled with a 20% PVDF solution. After evaporation of the DMF solvent, a crystallized PVDF piezoelectric film became embedded within the LCE-M3 matrix. The actuation strain of the actuator maintains ~ 99% after PVDF padding (Fig. S16). The novel direction of the PVDF film is defined as the effective polarization direction, enabling the piezoelectric film to exhibit d_31_ effect under the uniaxial actuation stress from LCE-M3. As shown in Fig. 5c, cyclic open-circuit potential (*V*_*OC*_) signals are triggered by the reversible deformation of LCE-M3 during heating periods. Notably, a significant discrepancy in *V*_*OC*_ is observed when the temperature stimulus increases from 60 to 80 °C. As the temperature of the actuator is close to its *T*_*ni*_, partial deformation results in a light increase in *V*_*OC*_. When the temperature is sufficiently high to induce complete deformation of the LCE-M3, a *V*_*OC*_ higher than that under 60 °C is observed. A stepwise increase in *V*_*OC*_ generated by the device in response to different temperature stimuli is shown in Fig. S17. The VOC levels under 70 and 80 °C exhibit similarity due to the same contraction condition of LCE-M at isotropic phase. This observation suggests the dependence of *V*_*OC*_ on the extent of deformation.

The *V*_*OC*_ signal is synchronized with the EM driven contraction of LCE-M3 actuator, as shown in Fig. 5d, e. Combining the actuation stress of LCE-M3 with the *V*_*OC*_ signal, the piezoelectric voltage raises along with the increasing actuation stress and reaches a peak point when the LCE-M actuator achieves full contraction in isotropic phase. The period of voltage accumulation in accordance with that of increasing actuation stress is slightly longer than the deformation period observed from strain variation due to the hysteresis of deformation (Fig. 5f). Subsequently, the voltage drops from its peak value as the actuation is completed. Linear correlation between the collected *V*_*OC*_ and actuation stress is demonstrated in Fig. 5g, where the linearly coefficient (R^2^) of 0.990 indicates that *V*_*OC*_ originates from piezoelectric output acting on LCE-M composite contraction [55]. This original strategy for self-powered actuation sensing utilizes energy conversion from mechanical to electrical through an EM sensitive LCE-M soft actuator, thereby enhancing intelligence in EM devices.

## Conclusions

The fabrication of an efficient electromagnetic dissipative structure is achieved through the hybridization of a smart polymer with MXene via stable intermolecular interactions. The electromagnetic response of this MXene-polymer hybrid structure is finely tuned by the various polarization factors, including space-charge polarization, orientation polarization and interfacial polarization. Based on these factors, the hybridized polymer demonstrates sensitive remote driving capabilities. The self-adaptive deformation in response to microwave stimulus can be activated within ~ 10 s due to the heat generated from electromagnetic attenuation. Furthermore, by incorporating an ultra-low content of MXene, the sensitization effect is achieved while minimizing any adverse impact on mechanical properties. Consequently, a self-powered motion-sensing strategy is prototyped to improve the intelligence of LCE-M-based actuation device further. Real-time feedback voltage is obtained through inherent mechanical–electrical energy conversion in an embedded piezoelectric polymer. This work presents a distinctive design for EM energy dissipative structures in hybrid polymers that can advance the research on miniaturized intelligent soft materials and devices.

## Supplementary Information

Below is the link to the electronic supplementary material.

Supplementary file1 (DOCX 2014 KB)
