# Supplementary material for: MXene Hybridized Polymer with Enhanced Electromagnetic Energy Harvest for Sensitized Microwave Actuation and Self-Powered Motion Sensing
Source: Nanomicro Lett. 2024 Nov 18;17:65. doi: 10.1007/s40820-024-01578-z (PMC11573944; doi:10.1007/s40820-024-01578-z)
Supplement: Supplementary file 1 — Supplementary file1 (DOCX 2014 KB) [file 40820_2024_1578_MOESM1_ESM.docx]

Supporting Information for

**MXene Hybridized Polymer with Enhanced Electromagnetic Energy Harvest for Sensitized Microwave Actuation and Self-powered Motion Sensing**

Yu-Ze Wang^1^, Yu-Chang Wang^1, 2^, Ting-Ting Liu^1^, Quan-Liang Zhao^3^, Chen-Sha Li^4^, Mao-Sheng Cao^1,^ *

^1^ School of Materials Science and Engineering, Beijing Institute of Technology, Beijing 100081, P. R. China

^2^ School of Materials Science and Engineering, Peking University, Beijing 100871, P. R. China

^3^ School of Mechanical and Material Engineering, North China University of Technology, Beijing 100144, P. R. China

^4^ Key Laboratory of Functional Inorganic Material Chemistry, Ministry of Education of the People’s Republic of China, Heilongjiang University, Harbin 150080, P. R. China

* Corresponding authors. E-mail: [caomaosheng@bit.edu.cn](mailto:caomaosheng@bit.edu.cn) (Mao-Sheng Cao)

**S1 Calculations and Statistical Analysis**

**S1.1 First-principles calculation**

The first-principles plane-wave pseudopotential method was adopted. The difference charge density around vacancy and functional group sites was obtained by the CASTEP program with a function of generalized gradient approximation (GGA) and Perdew-Burke-Ernzerhof (PBE), a 3a × 3b × c supercell, a custom cutoff energy of 450 eV, a SCF tolerance of 2 × 10^−6^ eV/atom, a k-point Monkhorst-Pack grid of 3×3×1 and the vacuum layer of 20 Å were adopted.

**S1.2 Calculation of order parameters (S) based on 2D-WAXS results**

$S=\frac{3<{cos}^{2}\varphi>-1}{2}$ (S1)

$<{cos}^{2}\varphi>=\frac{\int_{0}^{\frac{\pi}{2}} I(\varphi)\sin\varphi{cos}^{2}\varphi d\varphi}{\int_{0}^{\frac{\pi}{2}} I\left( \varphi\right)sin\varphi d\varphi}$ (S2)

Where *I* is the intensity and *φ* is the azimuthal angle presented in Fig. 2c. $<{cos}^{2}\varphi>$ is the mean squared cosine of the angle between the direction of mesogen alignment and the axial direction of the actuator.

**S1.3 Evaluation of EM properties**

The real part (*ε’*) and imaginary part (*ε”*) of complex permittivity (*ε**) are defined by Debye Eqs. (S3 and S4).

$\varepsilon^{'}=\varepsilon_{\infty}+\frac{\varepsilon_{s}-\varepsilon_{\infty}}{1+\omega^{2}\tau^{2}}$ (S3)

$\varepsilon^{''}=\frac{\varepsilon_{s}-\varepsilon_{\infty}}{1+\omega^{2}\tau^{2}}\omega\tau+\frac{\sigma}{\omega\varepsilon_{0}}=\varepsilon_{p}^{''}+\varepsilon_{c}^{''}$ (S4)

where *ε_∞_* is the relative dielectric permittivity at infinite frequency and *εs* is the static permittivity; *ω* is the angular frequency; *τ* is the relaxation time; *σ* is the electric conductivity. According to Debye’s relaxation theory and circular arc law, classic relaxation process shall be reflected by the peak of ε″_p_ and a semicircle curve in Cole-Cole plot, which conform to Eq. S5.

${{(\varepsilon}^{'}-\frac{\varepsilon_{s}+\varepsilon_{\infty}}{2})}^{2}+{\varepsilon_{p}}^{2}={(\frac{\varepsilon_{s}-\varepsilon_{\infty}}{2})}^{2}$ (S5)

**S1.4 Calculation of** **ratio of converted EM energy to stored EM energy (ω_r_)**

$\omega_{r}=\frac{\varepsilon^{''}\varepsilon_{0}E_{0}^{2}+\mu^{''}\mu_{0}H_{0}^{2}}{\varepsilon^{'}\varepsilon_{0}E_{0}^{2}+\mu^{'}\mu_{0}H_{0}^{2}}$ (S6)

where ε_0_ and μ_0_ represent vacuum permittivity and permeability, respectively; E_0_ and H_0_ are the electric field intensity amplitude and magnetic field intensity amplitude of the EM wave, respectively.

*Calculation of heating rate (R_H_) of LCE-M actuators under microwave stimulus:*

$R_{h}=\frac{T_{ni}-T_{r}}{t_{1}}$ (S7)

where T*r* and T*ni* are room temperature and phase transition temperature, respectively; t_1_ is the response time.

**S1.5 Calculation of apparent EM energy harvest efficiency (AE_EM_)**

${AE}_{EM}=\frac{Q_{a}}{W_{mic}}$ (S8)

$Q_{a}=\int_{T_{r}}^{T_{ni}} C_{p}\left( T \right)dT \times m$ (S9)

$W_{mic}=P_{r} \times t_{1}$ (S10)

where *Q_a_* and *W_mic_* stand for necessary heat for activating an actuator and total output work from the microwave generator during activation, respectively; *T_r_* and *T_ni_* refer to room temperature and phase transition temperature, respectively; *C_p_* refers to the specific heat capacity of the actuator, which is a function of temperature (*T*) and is acquired from the DSC results; m is the mass of the actuator; *P_r_* is the rated output power of the microwave generator and t_1_ is the response time.

**S1.6 Calculation of work capacity (*W*) and power density (*P*)**

$W=\frac{F_{max}\Delta L}{V}$ (S11)

$P=\frac{W}{t_{2}}$ (S12)

where F*_max_* is the maximum actuation stress; ΔL refers to the length variation corresponding to 90% of the maximum actuation strain, which conform to the identification of deformation period; t_2_ is the deformation period.

**S1.7 Pre-processing of data**

Irradiation time in all actuation tests is normalized to start at the moment of turning on the energy source.

**S1.8 Data presentation**

Error bars stand for standard deviation in bar charts (mean ± SD). Sample size for each statistical analysis is contained in the corresponding Figure legend.

**S2 Supplementary Figures and Tables**


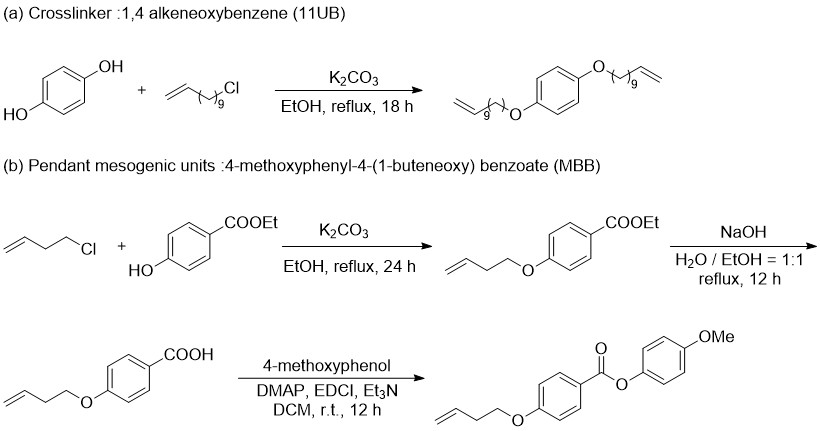


**Fig. S1** Synthetic route of crosslinker of 11UB and mesogenic unit of MBB


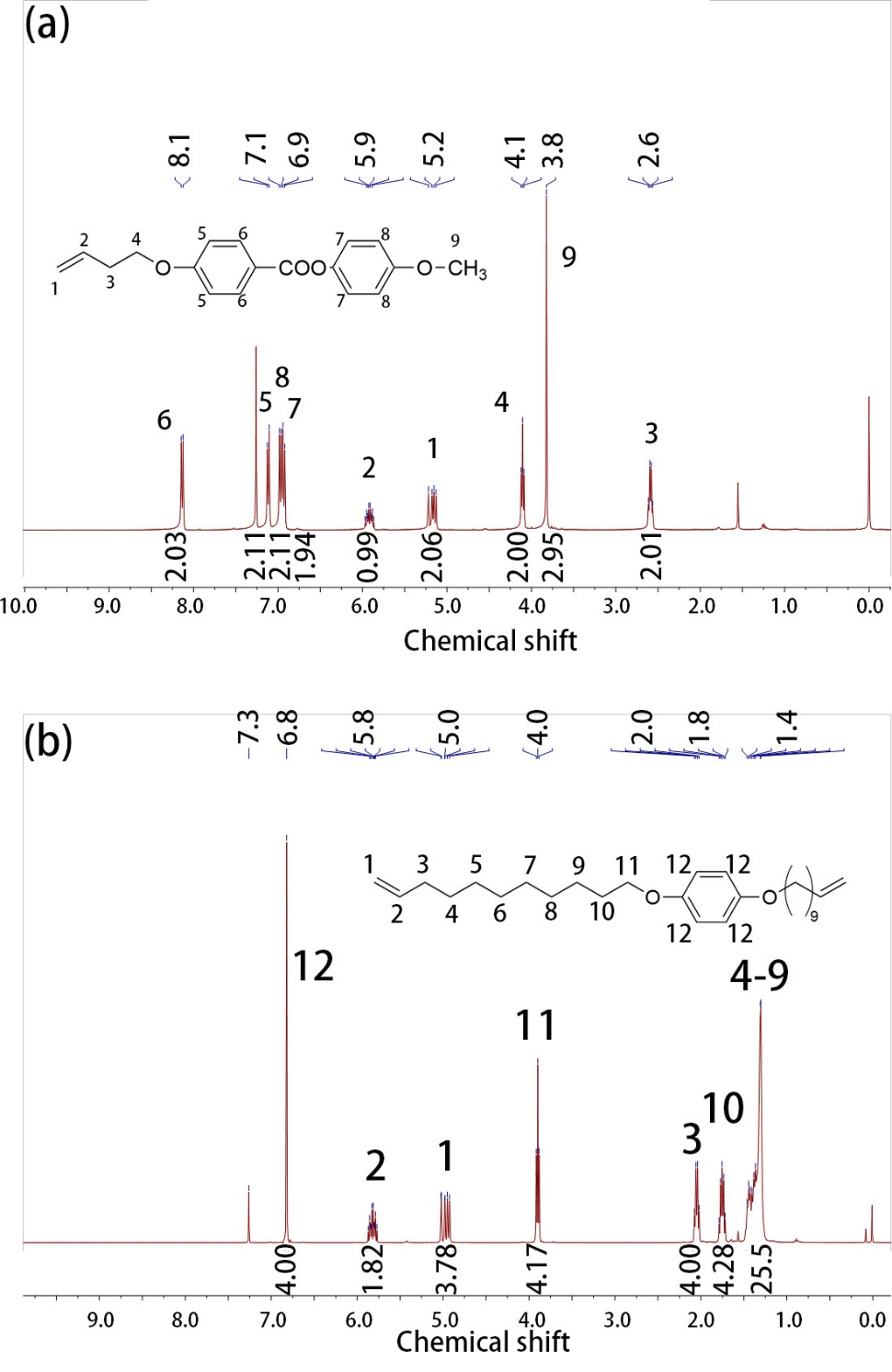


**Fig. S2** H-NMR spectroscopy results of the as prepared building blocks. (a) MBB; (b) 11UB


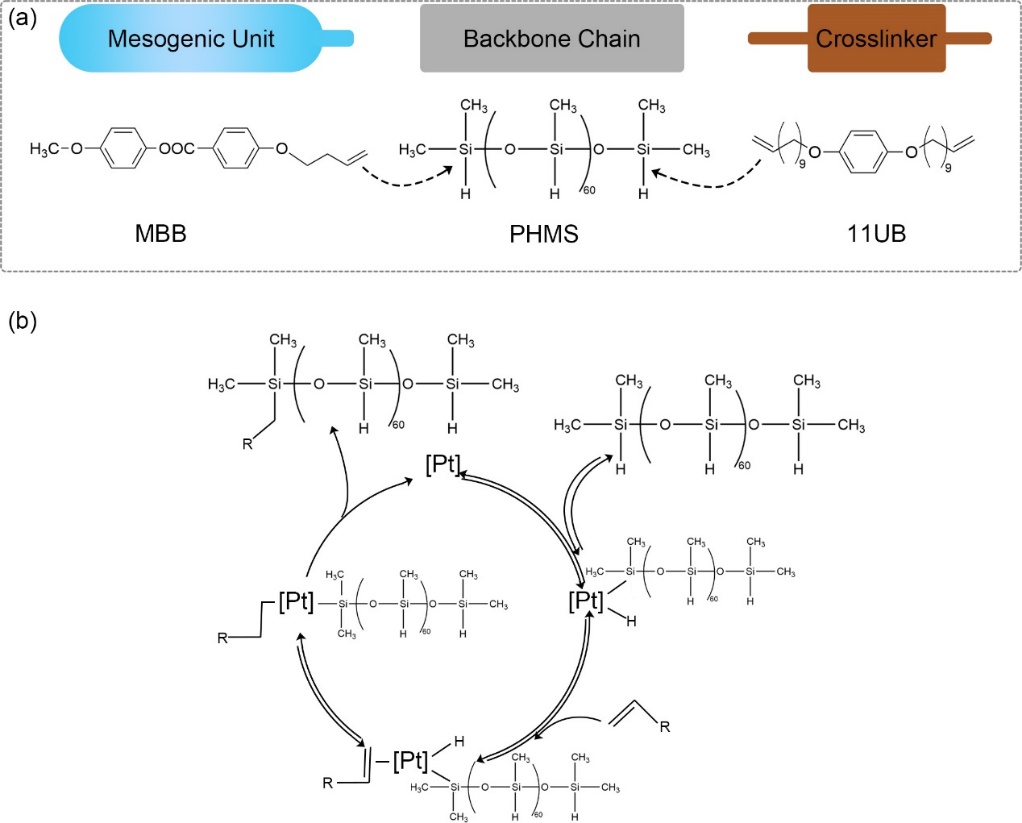


**Fig. S3** Reaction pathway of Pt catalized Si-H addition in the crosslinking process

**
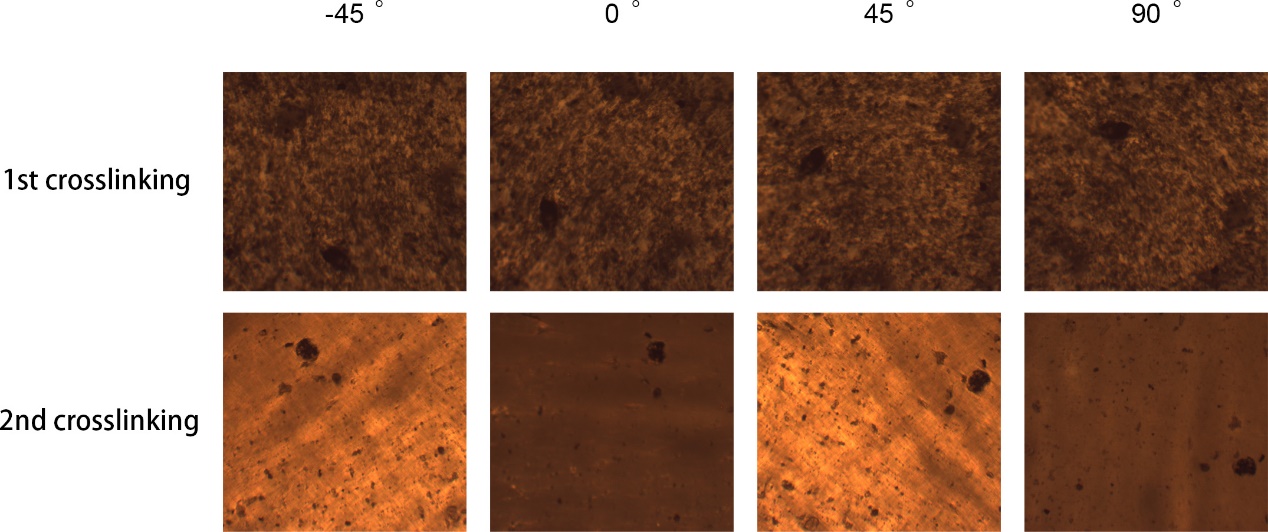
**

**Fig. S4** POM images of the polydomain conformation in LCE matrix after primary crosslinking and the monodomain conformation after secondary crosslinking.


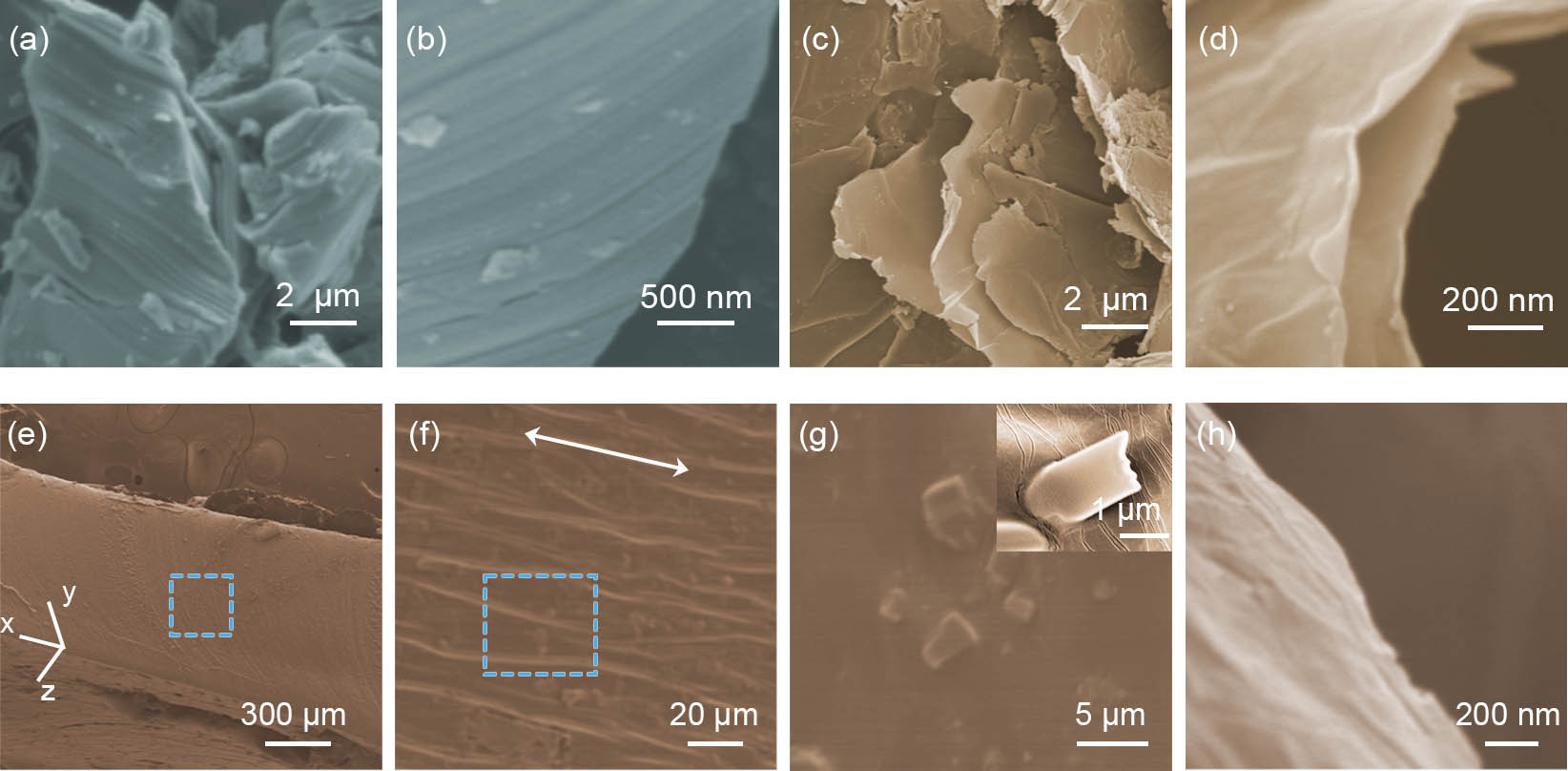


**Fig. S5** SEM images of MAX phase (**a-b**); MXene sheets (**c-d**); SEM images of LCE-M3 from cross-sectional view (**e-g**) and the edge area of a MXene sheet (**h**)


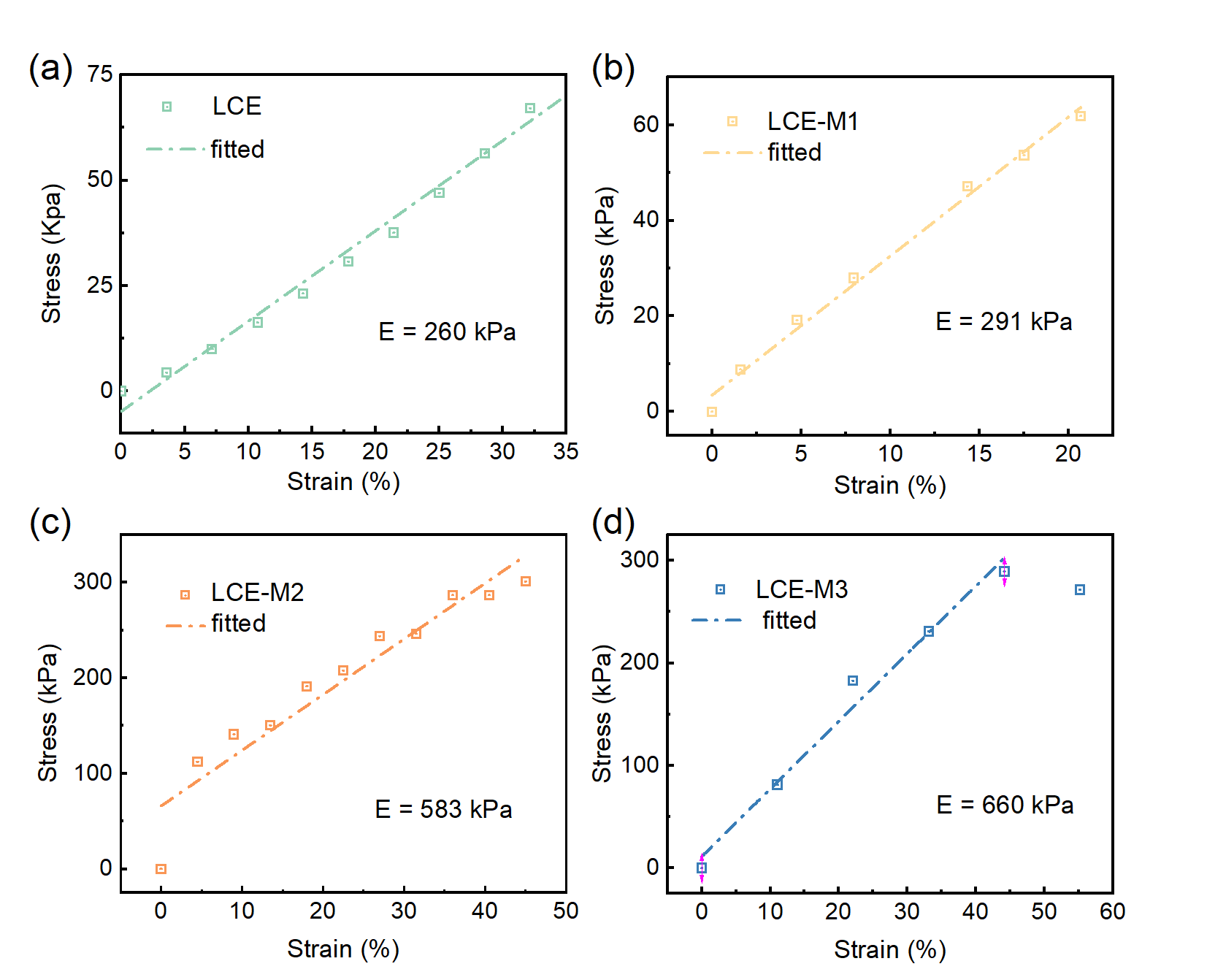


**Fig. S6** Stress-strain properties of LCE (**a**) LCE-M1 (**b**), LCE-M2 (**c**), LCE-M3 (**d**) and corresponding Young’s modulus


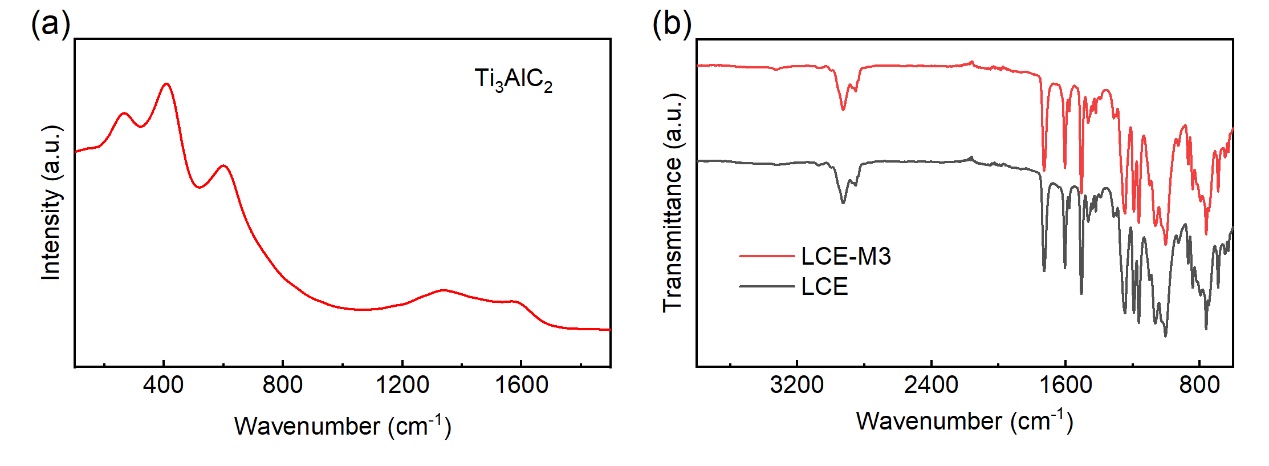


**Fig. S7** (**a**) Raman spectra of Ti_3_AlC_2_. (**b**) Comparation of ATR-FTIR spectra of LCE-M3 and LCE


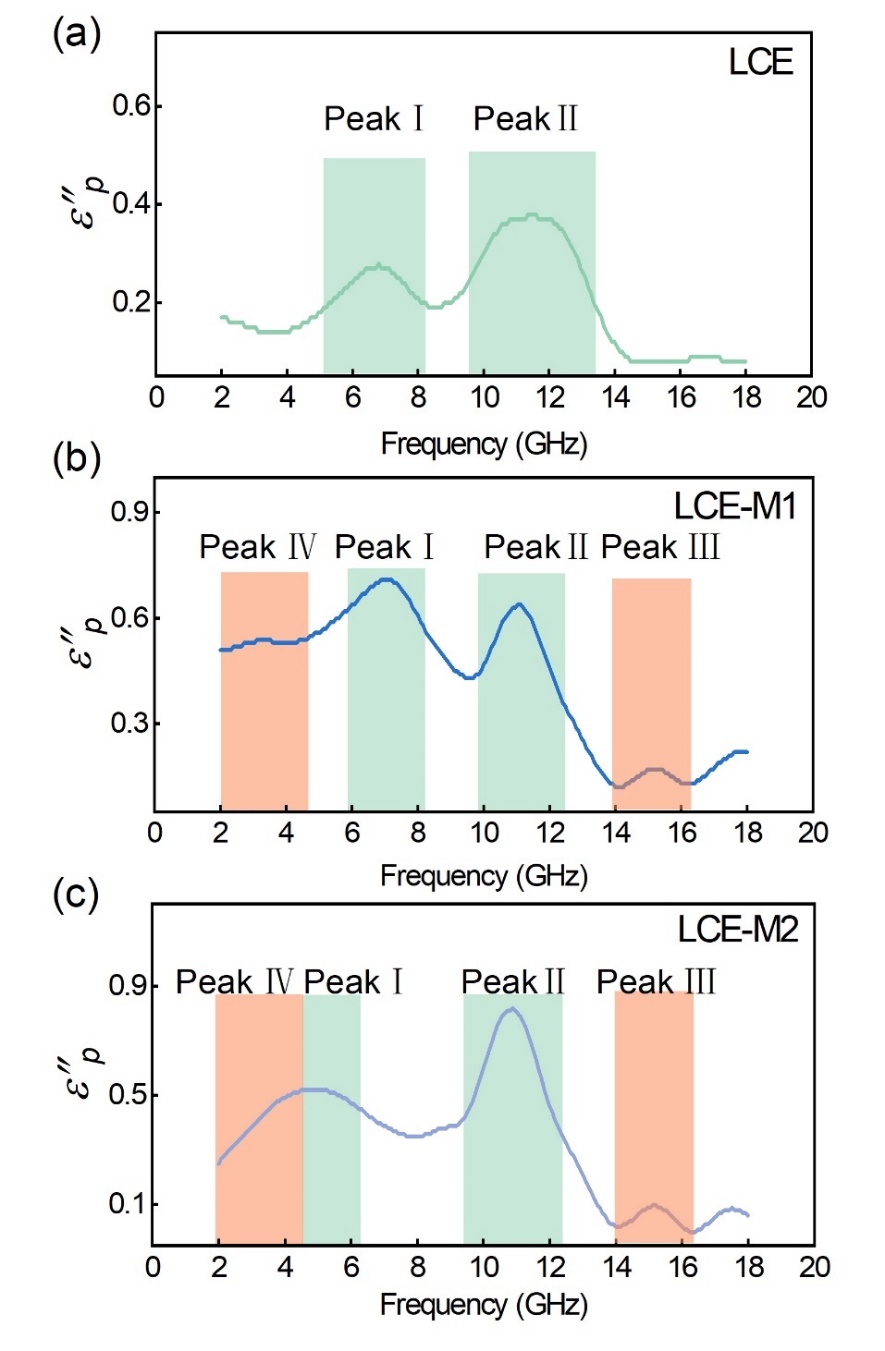


**Fig. S8** Relaxation peaks based on ε″_p_ of LCE (**a**), LCE-M1 (**b**) and LCE-M2 (**c**)


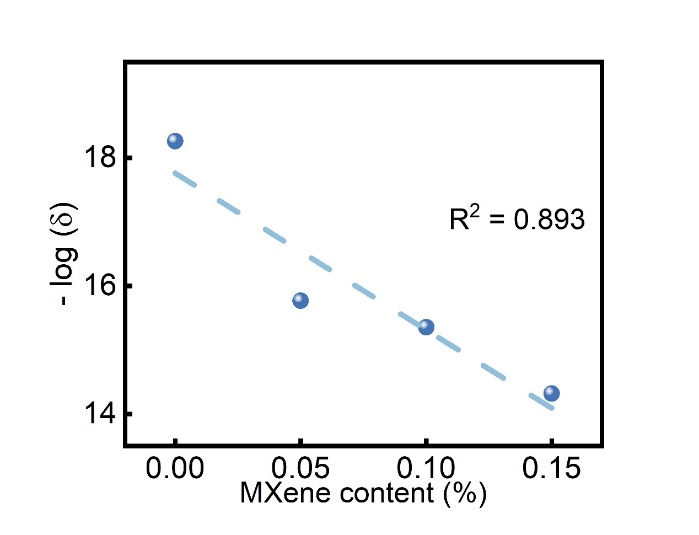


**Fig. S9** Linear fitting of the negative logarithm of conductivity versus loading content of MXene in LCE-M


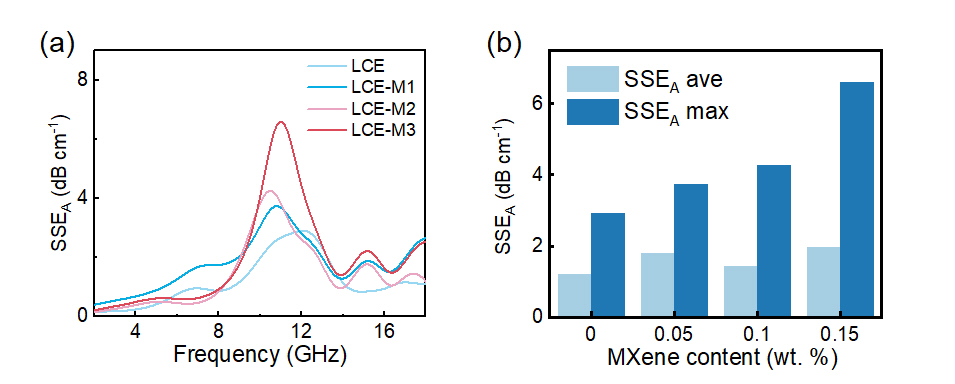


**Fig. S10** Specific absorption efficiency (SSE_A_) of LCE-M composites. (**a**) Frequency dependent SSE_A_ in the range of 2-18 GHz. (**b**) Average SSE_A_ and maximum SSE_A_ for various MXene content in LCE-M


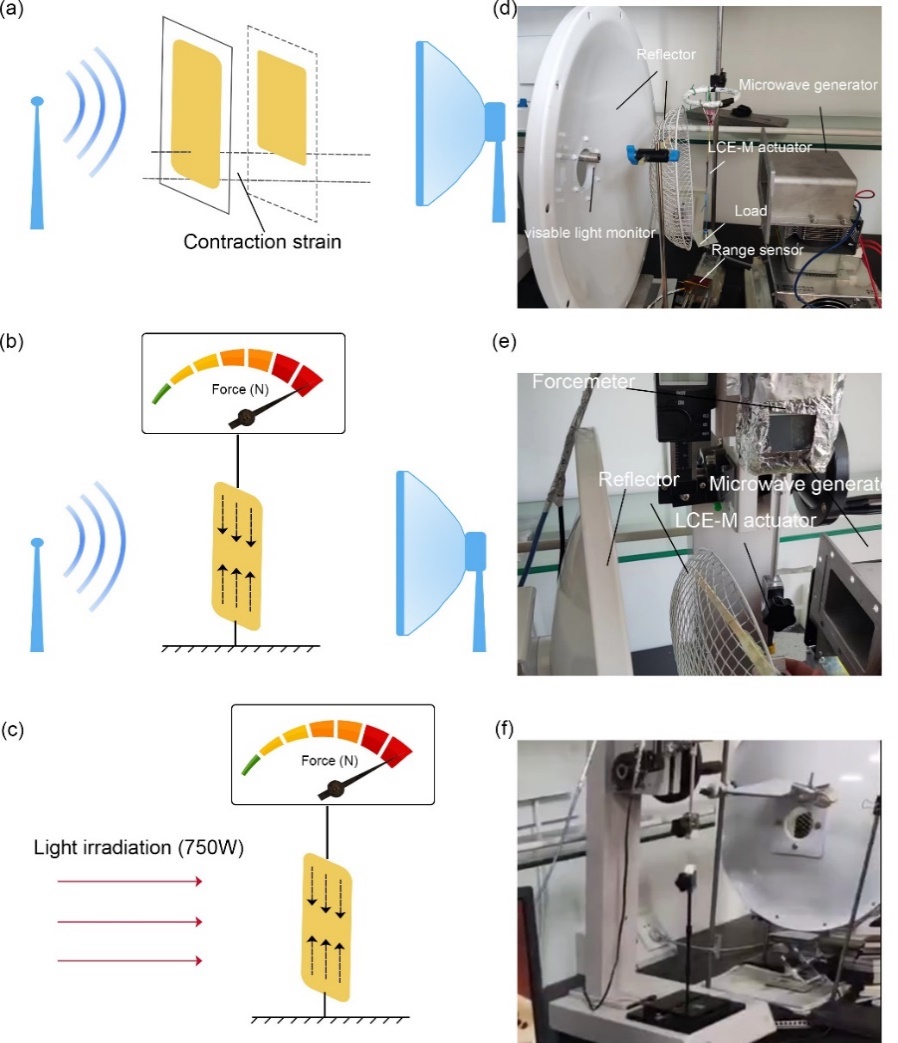


**Fig. S11** Deployment of the actuation test. (**a-c**) Schematic illustration of actuation strain testing (**a**), microwave actuation stress testing (**b**) and light actuation stress testing (**c**). (**d-f**) Photograph of the testing system corresponding to (**a-c**)


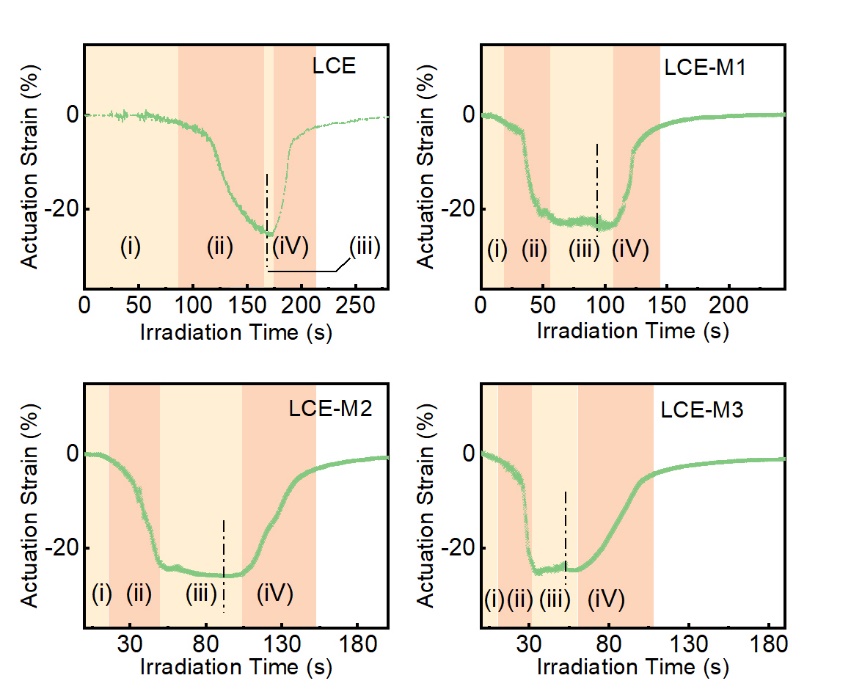


**Fig. S12** Actuation strain of the four actuators under microwave irradiation and their recovery in room temperature versus time. The dot-dash lines inset indicate the microwave being switched off


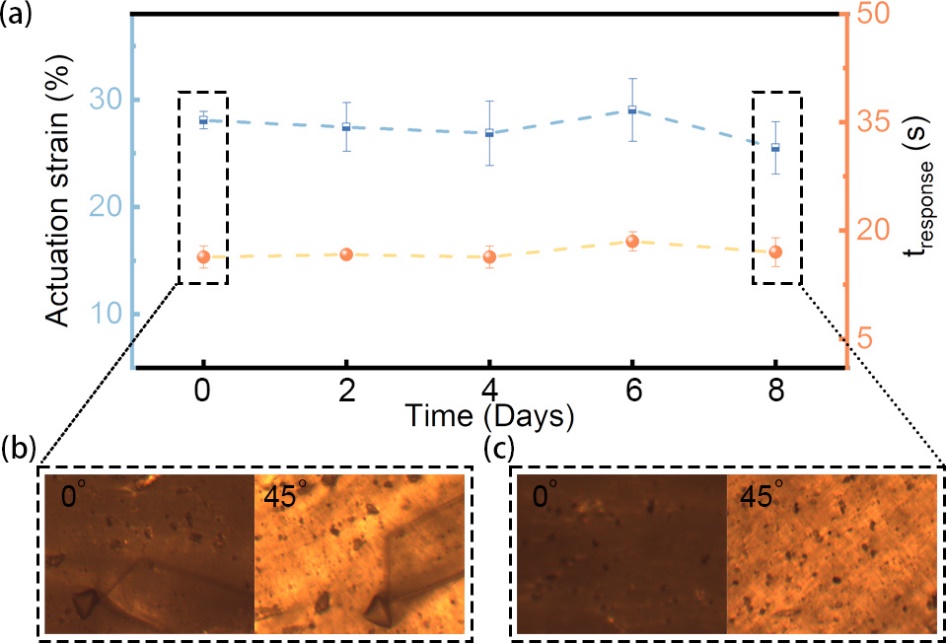


**Fig. S13** Stability of LCE-M3 actuator after microwave irradiation and storage for 8days. Sample size for each day is 5


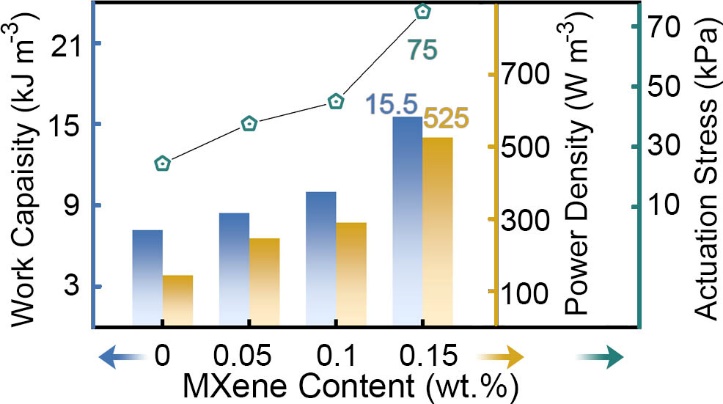


**Fig. S14** Actuation stress, work capacity and power density of LCE and LCE-M actuators


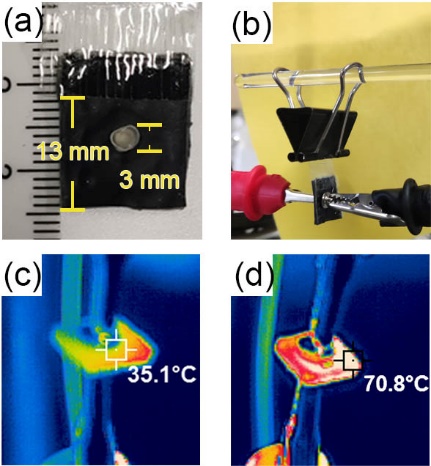


**Fig. S15** Photographs of **a**) prototype self-sensing artificial muscle and **b**) testing device collecting proprioceptive voltage signal. **c-d**) Infrared thermal images of PVDF@LCE-M3 before and after actuation


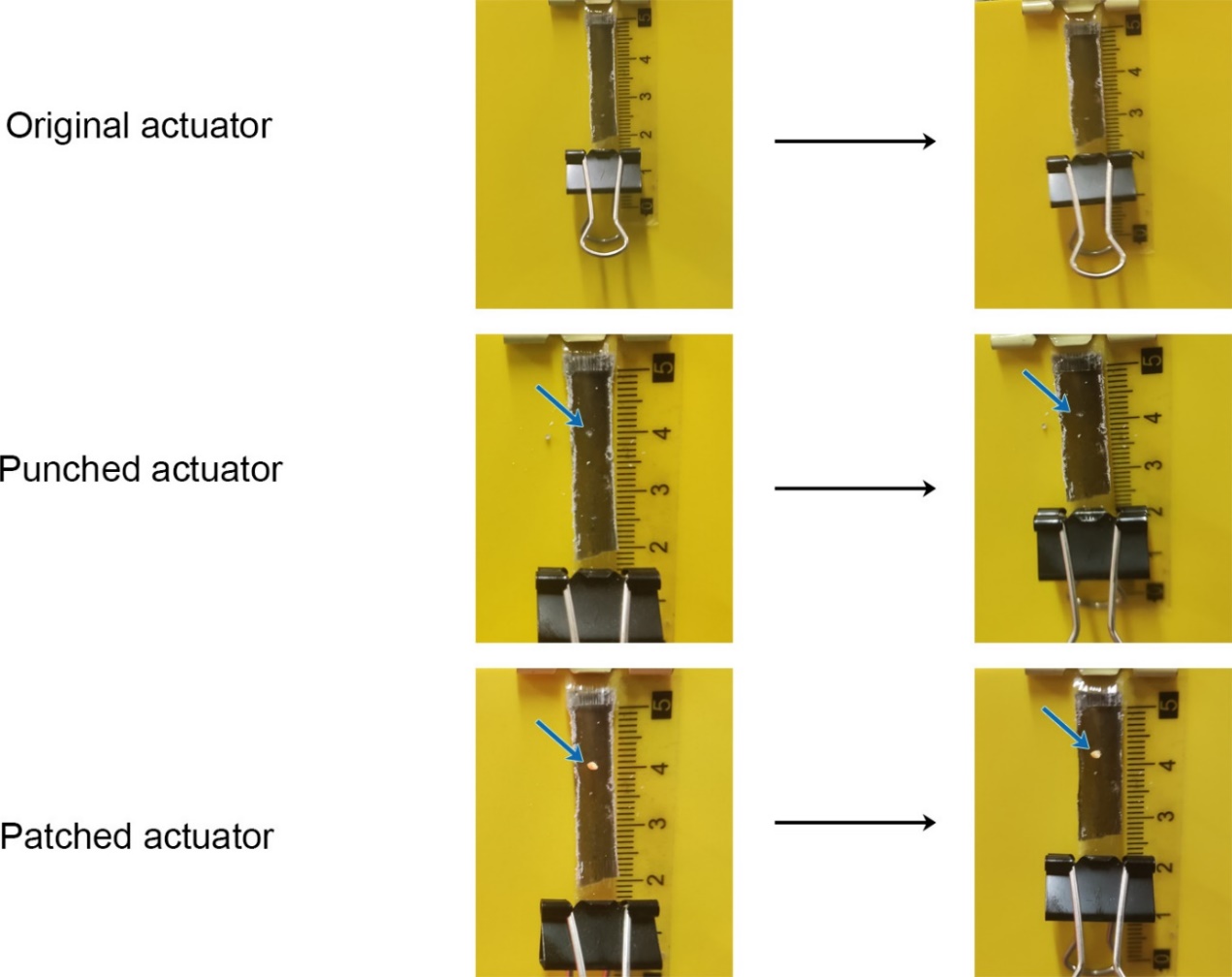


**Fig. S16** Photographs depicting the actuation strain of the LCE-M3, as well as its condition before and after patching


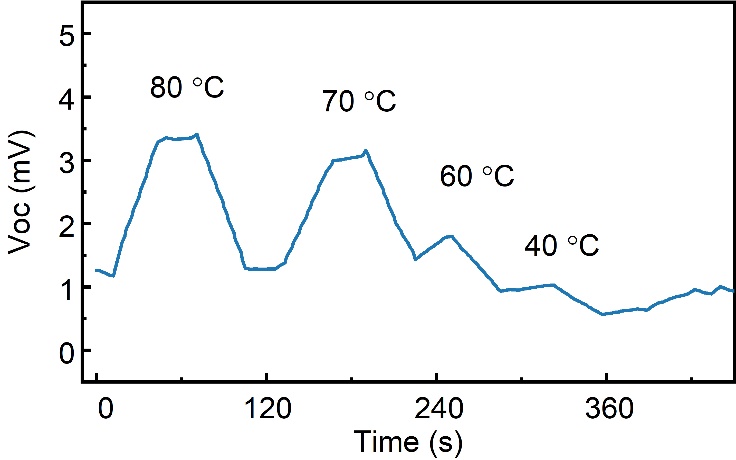


**Fig. S17** *V_OC_* output collected under different temperature

**Table S1** Comparison of microwave responsive shape memory polymers in actuation performance and energy conversion mechanism

| Material | Filler content (wt%) | Response time (s)* | Maximum strain (%) | Energy conversion mechanism | Refs. |
| --- | --- | --- | --- | --- | --- |
| iron@PLA | 45 | 30 | 43.2 | Condutive & magnetic loss | [S1] |
| Graphite@PLA | 15 | 5 | - | Not reported | [S2] |
| CNT@nylon 6 | 2 | 10 | 9.1 | Joule heating | [S3] |
| SiC@PVA | 1.5 | 45 | - | Dipole movement | [S4] |
| Expanded graphite@epoxy | 2.15 | 26 | - | Scattering & defect polarization | [S5] |
| rGO@LCE | 0.3 | 10 | 35.7 | Defect polarization | [S6] |
| LCE | - | 60 | 32.5 | Molecular polarization | [S7] |
| MXene-LCE | 0.15 | 10.3 | 25.5 | Multiple polarizaiton | This work |

* The specific definitions of response time vary in different literature, and can be significantly influenced by experimental settings. Here, response time is used to describe the period from microwave irradiation to forming significant deformation.

**Supplementary References**

1. T. Y. Koh, A. Sutradhar, Untethered selectively actuated microwave 4d printing through ferromagnetic PLA. Addit. Manuf. **56**, 102866 (2022). <https://doi.org/10.1016/j.addma.2022.102866>
2. S. C. An, Y. Lim, Y. C. Jun, Rapid and selective actuation of 3d-printed shape-memory composites via microwave heating. Sci. Rep. **13**, 18179 (2023). <https://doi.org/10.1038/s41598-023-45519-z>
3. S. Aziz, B. Villacorta, S. Naficy, B. Salahuddin, S. Gao et al., A microwave powered polymeric artificial muscle. Appl. Mater. Today **23**, 101021 (2021). <https://doi.org/10.1016/j.apmt.2021.101021>
4. H. Y. Du, Z. Song, J. J. Wang, Z. H. Liang, Y. H. Shen et al., Microwave-induced shape-memory effect of silicon carbide/poly(vinyl alcohol) composite. Sens. Actuator A Phys. **228**, 1-8 (2015). <https://doi.org/10.1016/j.sna.2015.01.012>
5. L. Chen, Y. Liu, J. Leng, Microwave responsive epoxy nanocomposites reinforced by carbon nanomaterials of different dimensions. J. Appl. Polym. Sci. **135**, 45676 (2018). <https://doi.org/10.1002/app.45676>
6. Y. C. Wang, Y. Z. Wang, J. C. Shu, W. Q. Cao, C. S. Li et al., Graphene implanted shape memory polymers with dielectric gene dominated highly efficient microwave drive. Adv. Funct. Mater. **33,** 2303560 (2023). <https://doi.org/10.1002/adfm.202303560>
7. X. Wang, Y. Wang, X. Wang, H. Niu, B. Ridi et al., A study of the microwave actuation of a liquid crystalline elastomer. Soft Matter **16**, 7332-7341 (2020). <https://doi.org/10.1039/d0sm00493f>
